# Éducation thérapeutique au Mali : un atelier de formation pour soignants et jeunes diabétiques de type 1

**DOI:** 10.48327/MTSIBULLETIN.2021.136

**Published:** 2021-08-23

**Authors:** A. Togo, M. Minkailou, P. Trebuchet, D. Sylla, A.T. Sidibé, S. Besançon, M. Nguemeni, T. Signe, B. Lab, M. Castellsague

**Affiliations:** 1 Service d'endocrinologie et de diabétologie, Hôpital du Mali, Yirimadio, Bamako, Mali; 2 Santé Diabète, Délégation au Mali, Bamako, Mali; 3 Hôpitaux Universitaires de Genève, Genève, Suisse

**Keywords:** Diabète, Diabète de type 1, Éducation thérapeutique du patient, Mali, Diabetes, Type 1 diabetes, Therapeutic Patient Education, Mali

## Abstract

En mars 2021, dans le cadre d'un partenariat entre les Hôpitaux Universitaires de Genève (HUG), l'Organisation Santé Diabète et le ministère de la Santé du Mali, une formation de 4 jours sous forme d'ateliers a été organisée avec l'objectif de renforcer les compétences des soignants en matière d’éducation thérapeutique du patient (ETP) destinée aux enfants et jeunes diabétiques de type 1. Pour assurer l'interprofessionnalité, des binômes médico-infirmiers travaillant déjà ensemble au sein des structures de santé de 7 régions du Mali ont participé à la formation. Au-delà de la complémentarité entre les professionnels, il s'agit de s'adapter aux réalités du système de santé malien. Toutes les activités étaient co-animés par des binômes de professionnels médecin et infirmier spécialisés en diabétologie et en ETP. Au total 30 professionnels, ont participé à la formation qui s'est déroulée en 2 jours de cours et 2 jours de camp avec des enfants diabétiques de type 1 et leur entourage. Pour renforcer la capacité des soignants à pratiquer l'ETP, nous avons choisi des approches innovantes avec des activités permettant la mise en pratique immédiate des compétences apprises avec divers outils pédagogiques. Les bénéficiaires étant non seulement les professionnels, mais aussi les enfants et adolescents et leur entourage, sans oublier les formateurs.

## Abstract (en)

In March 2021, in the framework of a partnership between the Geneva University Hospitals (HUG), the Santé Diabète Organization and the Ministry of Health of Mali, a 4-day workshop training course was organized with the objective of strengthening the skills of caregivers in Therapeutic Patient Education (TPE) for children and young people with type 1 diabetes. To ensure interprofessionalism, physician-nurse pairs already working together in health structures in 7 regions of Mali participated in the training. Beyond complementarity between health professionals, the aim was to cope with the realities of the Malian health system. All activities were co-chaired by physician-nurse pairs, specialized in diabetes and in TPE. A total of 30 professionals took part in the training, which consisted of 2 days of classroom training and 2 days of hands-on training with type 1 diabetic children and their families. To build the caregivers capacity in TPE, we chose innovative approaches with activities allowing the immediate application of skills learned with various pedagogical tools. The beneficiaries are not only the professionals, but also the children, teenagers and their families, including the trainers.

## Concept de l’éducation thérapeutique du patient (ETP)

L'apport de l'ETP dans la prise en charge des maladies chroniques a été largement démontré^1^ dans diverses maladies (diabète, asthme, insuffisance cardiaque obésité, infection à VIH), notamment en améliorant l'adhésion au traitement, en diminuant les complications et en améliorant la qualité de vie.

**Fig. 1:**
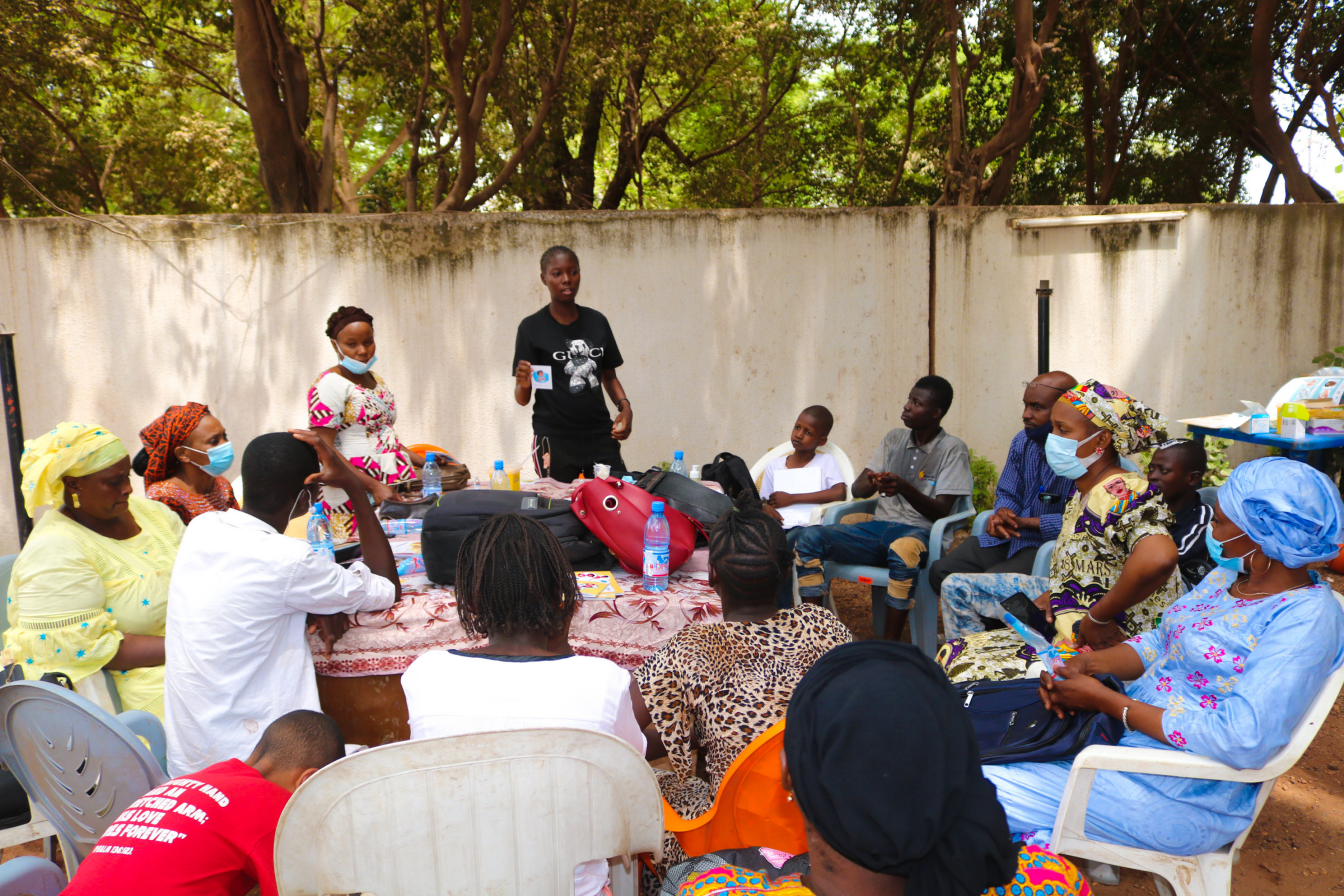
Atelier de formation pour les soignants, mars 2021 Training workshop for caregivers, March 2021

Selon la définition proposée par l'OMS « L’éducation thérapeutique doit permettre aux malades d'acquérir et de maintenir des compétences qui leur permettent de gérer de manière optimale leur traitement afin d'arriver à un équilibre entre leur vie et leur maladie. C'est un processus continu, qui fait partie intégrante des soins médicaux. (…) L'ETP est conçue pour aider les patients et leurs familles à comprendre la maladie et leur traitement, à coopérer avec les soignants, à vivre sainement et maintenir ou améliorer leur qualité de vie. » (OMS, 1988, https://apps.who.int/iris/bitstream/handle/10665/107997/E93849.pdf).

S'adapter à chaque patient, s'appuyer sur les connaissances et l'expérience personnelle, utiliser un langage simple et accessible, susciter l'interrogation et répondre à la demande du patient, ne pas utiliser la peur pour convaincre au risque de faire face à un rejet du type « vu les complications, est ce que cela vaut le coup de m'investir? », valoriser les progrès atteints par le patient qui va apprendre en faisant (principe du droit à l'erreur), ne pas juger, sont autant de principes qui s'imposent dans une ETP de qualité.

De plus, l'ETP vise à améliorer la gestion de la maladie au long cours, ce qui implique des changements de comportements tels que la prise des médicaments, les mesures d'auto-surveillance, les modifications d'habitudes alimentaires ou la mise en place d'une activité physique régulière qui sont difficiles à maintenir à long terme. Le travail motivationnel fait ainsi partie intégrante du processus d’éducation thérapeutique.

Dans le projet d'accompagner un individu porteur d'une malade chronique, l'ETP réinterroge le rôle du soignant en proposant un changement de paradigme.

L'ETP requiert de réunir des compétences multiples pour pouvoir appréhender la réalité complexe et toujours singulière des patients. Ces compétences peuvent être regroupées en 4 grands domaines: biomédicales, psychosociales, pédagogiques, philosophiques.

## Approche par les hôpitaux universitaires de genève (hug) de l'etp

En 1975, Le Professeur Jean Philippe Assal a créé la première Unité d'Enseignement Thérapeutique pour Diabétiques; avec la psychologue Anne Lacroix ils s'entourent d'une équipe interdisciplinaire pour assurer une approche bio-psycho sociale^2^. Cette Unité propose des semaines et des journées d'enseignement aux personnes vivant avec une maladie chronique, dans le but de les rendre capables de gérer leur maladie et d'acquérir plus d'autonomie. Les soignants, soutenus par les pédagogues, sont devenus des apprenants dans l'art de faire de l'enseignement en individuel et en groupe. En 1983, le service devient Centre collaborateur OMS et centre de recherche en enseignement du diabète. En 1985, l'Unité devient Division d'enseignement thérapeutique pour maladies chroniques (diabète, obésité…) et c'est en 1998 que le premier diplôme de formation continue en ETP a été créé avec la Faculté de Médecine.

## Prise en charge du diabète au mali

Depuis plusieurs années, Santé Diabète, en étroit partenariat avec le ministère de la Santé, a soutenu la transformation du système de santé du pays autour des 6 éléments constitutifs du système de santé de l'OMS pour renforcer la prévention et la prise en charge du diabète. En 2004, au Mali, il y avait des limites très fortes dans le système de santé pour la prévention et la prise en charge du diabète. Mais, en 2019, après 15 ans d'actions, la situation a radicalement changé pour les capacités de prévention et de prise en charge du diabète mais aussi pour la qualité de la prise en charge des patients. En effet, en 2019, le Mali disposait d'un service d'endocrinologie et de diabétologie renforcé et d'une sous-unité de prise en charge du diabète de type 1 avec 20 spécialistes, 32 consultations-diabète opérationnelles dans 7 régions du Mali prenant en charge plus de 20 000 patients atteints de diabète de type 2 et 950 atteints de diabète de type 1, disponibilité pour chaque consultation-diabète de la plateforme technique, du matériel d'analyse, du matériel médical; une baisse de 48% des prix de l'insuline et une division du prix des antidiabétiques oraux par 10, la mise en place d'un programme d’éducation thérapeutique et de prévention et concernant les associations de patients diabétiques leur regroupement dans une organisation faîtière, la Fédération nationale des diabétiques du Mali (Fenadim). Il reste cependant encore de nombreux enjeux notamment le renforcement de l’éducation thérapeutique des enfants et jeunes adultes atteints de diabète de type 1.

## Atelier de formation pour soignants et jeunes diabétiques de type 1

En Afrique, dans la majorité des pays, l'espérance de vie pour un enfant atteint de diabète de type 1 est d'un an post diagnostic ce qui entraîne un très faible nombre d'enfants et jeunes adultes suivis dans les structures de santé. Le Mali fait partie des quelques pays qui ont réussi à développer une prise en charge du diabète de type 1 qui permet, aujourd'hui, d'avoir plus de 950 enfants et jeunes adultes suivis dans les structures de santé du Mali. Cette première réussite a été rendue possible par un soutien du programme *Life For A Child* pour l'accès à l'insuline avec, en parallèle, des premières formations des médecins référents diabète (https://pubmed.ncbi.nlm.nih.gov/33586301/). Le nombre de patients nécessite le développement d'une nouvelle phase pour renforcer et étendre cette prise en charge tout en renforçant la qualité de vie des patients. Pour ceci, Santé Diabète et ces partenaires internationaux comme les HUG ont développé une approche unique en Afrique passant par la formation approfondie des médecins référents diabète et un renforcement très important de l’éducation thérapeutique des patients.

Ainsi, en mars 2021, dans le cadre d'un partenariat entre les HUG, Santé Diabète et le ministère de la Santé malien, une formation sous forme d'ateliers a été organisée avec l'objectif de renforcer les compétences des soignants en matière d'ETP (ill 1). Pour assurer l'interdisciplinarité, des binômes médecin-infirmier travaillant déjà ensemble au sein d'une structure de santé ont participé à la formation. Au-delà de la complémentarité entre les professionnels, il s'agit de s'adapter aux réalités du système de santé malien. Hormis dans certaines structures à Bamako, la réalité est que les médecins sont très souvent amenés à s'absenter et sont mobilisés par des tâches autres que cliniques. D'où l'importance à ce qu'au sein de ces binômes, les deux professionnels soient mis sur un pied d’égalité en ce qui concerne la maitrise des principes essentiels de l'ETP. Au total 30 professionnels, médecins et infirmiers, venant de 7 régions différentes du Mali, ont participé à la formation qui s'est déroulé en 4 jours:

- 2 jours de formation;
- 2 jours de camp avec des enfants diabétiques de type 1 et leur entourage.

L'objectif principal de cette formation étant de renforcer la capacité des soignants à pratiquer l'ETP, nous avons choisi des approches innovantes comme par exemple: dès le premier jour il est demandé aux soignants d'effectuer une glycémie plusieurs fois par jour, de s'injecter avec des seringues à insuline (du NaCl) et de remplir le carnet de suivi en y rapportant toutes les informations utiles en lien avec la prise en charge du diabète (glycémie, alimentation, exercice physique …), ceci dans le but de faire ressentir aux soignants ce qu'une personne diabétique vit au quotidien. Cette approche dénommée « vivre comme un diabétique » déstabilise tout d'abord les participants habitués à des formats d'enseignement plus classiques (ex: cours magistraux). Mais, rapidement, cela génère de l'intérêt et des interrogations sur les techniques permettant de transmettre aux jeunes diabétiques les bonnes pratiques et d'acquérir les compétences afin de mieux vivre avec leur maladie.

Les deux journées appelés « Camps » pour enfants et jeunes diabétiques ont comme objectif la mise en pratique des techniques en ETP apprises les jours précédents. Ainsi le 3^e^ jour, les soignants ont la responsabilité d'encadrer un groupe de 20 adolescents diabétiques de type1 et d'animer les 4 ateliers sur les thèmes retenus:

1. la mesure de la glycémie et l'interprétation du résultat (hyper et hypoglycémie);
2. l'utilisation des bandelettes urinaires;
3. les techniques d'injection de l'insuline; et
4. les conseils diététiques et la gestion des exercices physiques.

Le 4^e^ jour les professionnels vont encadrer les 20 adolescents afin que ce soit les jeunes qui animent les 4 ateliers cette fois destinés à 20 enfants de moins de 15 ans, accompagnés d'un parent. Cela permet aux adolescents de transmettre leurs compétences aux plus jeunes et de les valoriser.

Au cours de la formation, le matériel pédagogique, créé par les équipes de Santé Diabète et de la Société malienne d'endocrinologie et de diabétologie (Somed), validé par le ministère de la Santé, et se présentant sous des formes adaptées aux différents publics, est présenté et utilisé en groupes de 6 à 8 soignants. Chaque binôme médecin-infirmier reçoit, en fin de formation, une valise contenant ces outils pédagogiques ainsi qu'une clef USB avec l'ensemble des cours de la formation.

Tous les cours étaient co-animés par des duos de professionnels médecin et infirmier spécialisés en diabétologie et ETP. Une évaluation pré et post formation a révélé l'amélioration des connaissances des participants. Ces derniers ont évalué les 4 jours de formation quant à l'organisation, le contenu ainsi que le format et la méthodologie.

## Conclusion et perspectives

Ce modèle de formation a permis un apprentissage avec la mise en pratique immédiate pour mieux intégrer les notions d'ETP et les outils pédagogiques. C'est une formation qui peut apporter des bénéfices aux professionnels qui y participent, ainsi qu'aux enfants et adolescents et leur entourage, sans oublier les formateurs qui doivent constamment s'adapter aux besoins et contexte de chacun. Le succès de ces ateliers d’éducation thérapeutique pour les enfants et jeunes adultes atteints de diabète de type 1 a permis de valider la méthodologie développée par les médecins spécialistes, les équipes des HUG et de l'ONG Santé Diabète.

De nouvelles journées d'ETP seront organisées à Bamako pour toucher une majorité d'enfants et de jeunes adultes atteint de diabète de type 1. Elles seront ensuite décentralisées dans les différentes régions du Mali prenant en charge ces patients.

En parallèle, il sera intéressant de continuer avec un accompagnement des professionnels sur leur lieu de travail, afin de mieux percevoir les difficultés rencontrées et de participer à l'amélioration de ces pratiques d'ETP adaptées aux divers contextes.

## Conflits D'intérêts

Les auteurs ne déclarent aucun conflit d'intérêt.
